# Investigating the effect of sesame meal replacement for soybean meal in diets with different levels of calcium and phytase enzyme in broiler chickens

**DOI:** 10.1002/vms3.1379

**Published:** 2024-02-15

**Authors:** Fatemeh Aziz‐Aliabadi, Fariba Amirzadeh‐Garou, Ahmad Hassanabadi, Hadi Noruzi

**Affiliations:** ^1^ Department of Animal Science Faculty of Agriculture Ferdowsi University of Mashhad Mashhad Iran

**Keywords:** broiler, calcium, phytase, sesame meal, soybean meal, tibia

## Abstract

**Background:**

As a major part of the cost of broiler farms is related to their feed, breeders are looking for diets at a reasonable price. One of these affordable ingredients is sesame meal (SM), which can be used to replace soybean meal (SBM) in diets.

**Objectives:**

This study aims to investigate the effect of replacing SBM with SM in diets with different levels of calcium (Ca) and phytase (Phy) during the grower (11–24 days), finisher (25–42 days) and whole phases (11–42 days) on the growth performance, carcass characteristics and blood serum parameters of broiler chickens.

**Methods:**

A total of 600 1‐day‐old Ross 308 broiler male chicks were randomly dispensed to 12 dietary treatments with five replicates (10 birds per replicate) based on a completely randomized design in a factorial arrangement of 3 × 2 × 2 with 3 levels of SM (0%, 10% and 20%), 2 levels of Ca (standard (0.87% and 0.79% for grower and finisher phases, respectively) and 0.2% higher than the standard amount) and 2 levels of Phy (0 and 700 FTU/kg diet).

**Results:**

During the study, the significant effects of SM, Ca and Phy on the daily average body weight gain (DAWG) and daily feed intake (DFI) were observed, whereas for the feed conversion ratio, only the effect of SM was significant (*p* < 0.05). During the finisher and whole phases, the SM and Ca levels influenced the DAWG and DFI, whereas the interaction between Phy and Ca was also significant for DAWG (*p* < 0.05). The main effects of SM and Ca on the relative weights of breast, heart and liver were significant (*p* < 0.05). Application of 20% of SM significantly reduced serum Ca concentration (*p* < 0.05).

**Conclusion:**

Generally, the inclusion of more than 10% of SM in the broilers diet is not recommended. In addition, the use of Phy and Ca levels 0.2% higher than standard in broilers diet could improve the birds’ performance.

## Graphical abstract

Sesame meal is an appropriate substitute for soybean meal. Phytate in sesame meal binds minerals including calcium and reduces its availability. Use of phytase enzyme and calcium level 0.2% higher than standard in broilers diet improve their performance.

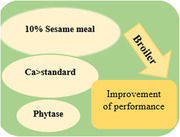

## 1 INTRODUCTION

Due to the high price of soybean meal (SBM), the use of other vegetable protein sources as an alternative to SBM in poultry feed has received much attention. Sesame (*Sesamum indicum L*.) is one of the native plants of Iran, which has been cultivated in tropical and subtropical regions of the country for centuries. This seed has approximately 50% oil, 25.5% crude protein (CP) and 5.5% ash. Sesame meal (SM) is the remaining product after oil extraction, which has about 40%–60% protein (Pan et al., 1992). In addition, SM is a rich source of minerals but its mineral availability is low due to high levels of oxalate and phytate (Ravindran & Blair, 1992). It has been reported that the SM is rich in sulphur‐containing amino acids, but compared to SBM, the content of lysine, isoleucine, leucine and valine is lower (Kaneko et al., 2002; Singh & Khanna, 1988).

SM has a significant amount of phytate, and this phytate reduces the availability of protein and minerals, including calcium (Ca), in the poultry diets (Farran, 2000). By using phytase (Phy) enzyme, the dependence on the use of inorganic phosphorus (P) sources can be reduced. By hydrolysing phytate complexes, this enzyme can release P, minerals, amino acids and starch and make them available for absorption (Ravindran et al., 1999; Zwart, 2006). The amount of Ca in SM varies from 0.91% to 2.80% depending on the meal production method (mechanical or solvent extraction) (Abbasi et al., 2022; Babiker, 2012; Hajimohammadi et al., 2020;). However, only 53% of Ca in SM is available (Mamputu & Buhr, 1995). Bell et al. (1990) reported that SM could replace up to 25% in broilers diets. Various studies (Pan et al., 1992; Kang et al., 1999) have suggested that the most suitable level of SM replacement for SBM is 15% or less. Al Harthi and El‐Deek (2009) reported that the body weight and daily weight gain (DWG) of broilers fed with a diet containing 5% SM was significantly higher than the control group. The addition of Phy in the group containing 10% SM made the performance of this group higher than the control. It has been stated that SM can be included up to 20% in the laying hens’ diet without having a detrimental effect on the growth performance (Yamauchi et al., 2006). Considering the price of SBM and the low accessibility of Ca in SM, the purpose of this study was to investigate the possibility of replacing SBM with SM along with the use of Phy and a higher level of Ca (0.2% higher than the standard amount).

## 2 MATERIALS AND METHODS

### 2.1 Birds, diets and housing

The Animal Care Committee of the Ferdowsi University of Mashhad, Mashhad, Iran approved all protocols.

Six hundred 1‐day‐old Ross 308 broiler male chicks were provided from a commercial hatchery (Simorgh Company) and assigned to 60 pens and nurtured for 42 days. Twelve experimental diets as a 3 × 2 × 2 factorial arrangement with three levels of SM (0%, 10% and 20%), two levels of Ca (standard (0.87% and 0.79%) and 0.2% higher than the standard amount [1.04% and 0.955%]) and two levels Phy (0 and 700 FTU/kg diet) were fed to the birds during the grower (11–24 days of age) and finisher (25–42 days of age) periods. Five replicates were considered for each treatment and each replicate contained 10 birds. All birds were fed with a standard diet until the age of 10 days and then received experimental treatments at the age of 11 days. All diets were formulated based on the Ross 308 nutrition guidelines (Aviagen, 2017). The ingredients and nutrient composition of diets are highlighted in Tables 1 and 2 (NRC, 1994). SM was prepared from the local sesame of Kalat Nader‐Iran by cold press extraction method. The SM was analysed to determine the amount of dry matter, CP, crude fibre, ash, Ca and P (Association of Official's Analytical Chemists [AOAC], 2005) (Table 3). The temperature of the rearing place was set at 32°C in the first 3 days and reduced by 3°C each week to attain 21°C and remained constant until the termination of the study. Relative dampness was between 60% and 70%. The first 2 days were set with a 24‐h light schedule followed by 18 h of light and 6 h of darkness. The birds had access to feed and water during the whole experimental period.

**TABLE 1:** Ingredients and nutrient composition of the starter and grower diets (as‐fed basis).

		Grower (11–24 days)					
	Starter (1–10 days)	Standard level of Ca			0.2% higher than the standard level of Ca		
Ingredients (%)	No SM	No SM	10% SM	20% SM	No SM	10% SM	20% SM
Corn	50.47	55.46	55.24	55.04	54.10	53.88	53.66
Soybean meal (44%)	42.96	37.12	28.67	20.23	37.39	28.95	20.51
Soybean oil	2.60	3.42	2.16	0.91	3.86	2.60	1.34
Calcium carbonate	1.32	1.23	1.35	1.46	1.38	1.49	1.61
Dicalcium phosphate	1.59	1.40	1.20	0.99	1.91	1.71	1.51
Common salt	0.29	0.22	0.22	0.22	0.22	0.22	0.22
Sodium bicarbonate	0.00	0.10	0.10	0.10	0.10	0.10	0.10
Vitamin premix^a^	0.25	0.25	0.25	0.25	0.25	0.25	0.25
Mineral premix^b^	0.25	0.25	0.25	0.25	0.25	0.25	0.25
l‐Lysine HCl	0.00	0.22	0.32	0.41	0.21	0.31	0.41
dl‐Methionine	0.27	0.33	0.24	0.14	0.33	0.24	0.14
Nutrient composition (%)							
Metabolizable energy (kcal/kg)	3000	3100	3100	3100	3100	3100	3100
Crude protein	23.00	21.50	21.50	21.50	21.50	21.50	21.50
Dig. methionine	0.63	0.62	0.62	0.61	0.62	0.62	0.61
Dig. Lysine	0.31	1.15	1.15	1.15	1.15	1.15	1.15
Dig. methionine + cystine	0.95	0.87	0.87	0.87	0.87	0.87	0.87
Dig. threonine	0.92	0.77	0.80	0.80	0.77	0.75	0.80
Calcium	0.96	0.87	0.87	0.87	1.04	1.04	1.04
Available phosphorus	0.48	0.43	0.43	0.43	0.52	0.52	0.52
Sodium	0.16	0.16	0.16	0.16	0.16	0.16	0.16
Potassium	1.28	1.16	1.06	0.96	1.16	1.06	0.96
Chlorine	0.22	0.22	0.24	0.29	0.22	0.24	0.26

*Note*: SM: sesame meal (36.72%).

Abbreviations: Ca, calcium; Dig, digestible, SM, sesame meal.

^a^ Provided the followings per kg of diet: vitamin A (trans‐retinyl acetate), 12,500 U; vitamin D3 (cholecalciferol), 5000 U; vitamin E (dl‐α tocopherol acetate), 80 U; vitamin K (menadione), 3.20 mg; riboflavin, 8.6 mg; pantothenic acid (d‐Ca pantothenate), 18.6 mg; pyridoxine (pyridoxine‐HCl), 4.86 mg; thiamine, 3.2 mg; vitamin B12 (cyanocobalamin), 0.02 mg; biotin, 0.25 mg; folic acid, 2.2 mg; nicotinic acid, 62.51 mg; choline chloride, 500 mg; ethoxyquin (antioxidant), 2.5 mg.

^b^ Provided the following per kg of diet: Fe, 20.23 mg; Zn, 110 mg; Mn, 120 mg; Cu, 16 mg; I, 1.25 mg; Se, 0.30 mg.

**TABLE 2:** Ingredients and nutrient composition of the finisher diets (as‐fed basis).

	Finisher (25–42 days)					
	Standard level of Ca			0.2% higher than the standard level of Ca		
Ingredients (%)	No SM	10% SM	20% SM	No SM	10% SM	20%SM
Corn	60.24	60.04	59.81	59.01	58.78	58.59
Soybean meal (44%)	31.68	23.23	14.79	31.93	23.49	15.04
Soybean oil	4.35	3.09	1.84	4.75	3.49	2.23
Calcium carbonate	1.15	1.26	1.38	1.28	1.40	1.51
Dicalcium phosphate	1.24	1.03	0.83	1.70	1.50	1.29
Common salt	0.22	0.22	0.22	0.22	0.22	0.22
Sodium bicarbonate	0.10	0.10	0.10	0.10	0.10	0.10
Vitamin premix^a^	0.25	0.25	0.25	0.25	0.25	0.25
Mineral premix^b^	0.25	0.25	0.25	0.25	0.25	0.25
l‐Lysine HCl	0.22	0.32	0.42	0.21	0.31	0.41
dl‐Methionine	0.30	0.21	0.11	0.30	0.21	0.11
Nutrient composition (%)						
Metabolizable energy (kcal/kg)	3200	3200	3200	3200	3200	3200
Crude protein	19.50	19.50	19.50	19.50	19.50	19.50
Dig. methionine	0.57	0.56	0.56	0.57	0.56	0.56
Dig. lysine	1.03	1.03	1.03	1.03	1.03	1.03
Dig. methionine + cystine	0.80	0.80	0.80	0.80	0.80	0.80
Dig. threonine	0.70	0.71	0.73	0.70	0.72	0.73
Calcium	0.79	0.79	0.79	0.95	0.95	0.95
Available phosphorus	0.39	0.39	0.39	0.47	0.47	0.47
Sodium	0.16	0.16	0.16	0.16	0.16	0.16
Potassium	1.04	0.94	0.84	1.04	0.94	0.84
Chlorine	0.22	0.24	0.26	0.22	0.24	0.26

*Note*: Sesame meal (36.72%).

Abbreviations: Ca, calcium; Dig, digestible; SM, sesame meal.

^a^ Provided the followings per kg of diet: vitamin A (trans‐retinyl acetate), 12,500 U; vitamin D3 (cholecalciferol), 5000 U; vitamin E (dl‐α tocopherol acetate), 80 U; vitamin K (menadione), 3.20 mg; riboflavin, 8.6 mg; pantothenic acid (d‐Ca pantothenate), 18.6 mg; pyridoxine (pyridoxine‐HCl), 4.86 mg; thiamine, 3.2 mg; vitamin B12 (cyanocobalamin), 0.02 mg; biotin, 0.25 mg; folic acid, 2.2 mg; nicotinic acid, 62.51 mg; choline chloride, 500 mg; ethoxyquin (antioxidant), 2.5 mg.

^b^ Provided the following per kg of diet: Fe, 20.23 mg; Zn, 110 mg; Mn, 120 mg; Cu, 16 mg; I, 1.25 mg; Se, 0.30 mg.

**TABLE 3:** Chemical composition of sesame meal.

Nutrients	Amount
Metabolizable energy (kcal/kg)	4265
Dry matter (%)	90.49
Crude protein (%)	36.72
Crude fibre (%)	8.82
Ash (%)	9.37
Calcium (%)	2.03
Phosphorus (%)	0.73

### 2.2 Growth performance

The birds in each pen were weighed collectively at 10, 24 and 42 days of age. The daily average body weight gain (DAWG) and daily feed intake (DFI) were determined for each pen. Feed intake was measured by subtracting the remaining feed from the offered feed in each pen during each study period. Feed conversion ratio (FCR) was corrected for mortality and represented as grams of feed consumed by all chickens in each pen divided by grams of body weight gain. Mortality for each pen was recorded daily.

### 2.3 Carcass characteristics

At 42 days of age, two birds per pen were selected, weighed and decapitated to measure carcass yield. The carcass, breast, thigh, wings, liver and heart were weighed separately. The results were expressed as a per cent of carcass.

### 2.4 Serum parameters

At 42 days of age, blood samples were obtained via the wing vein from two birds in each pen. Blood samples were centrifuged (2000 × *g* for 10 min), and serum was separated and then stored at −20°C until assayed for measuring biochemical parameters. Appropriate laboratory kits (Ziestchem kit, Zist Shimi Company) were used to determine triglycerides, total cholesterol, high‐density lipoprotein (HDL) cholesterol, Ca and P levels. Very low‐density lipoprotein (VLDL) cholesterol was determined by dividing triglyceride levels by 5, and low‐density lipoprotein (LDL) cholesterol was computed as the discrepancy amongst whole cholesterol and the composed condensations of HDL and VLDL (Friedewald et al., 1972).

### 2.5 Content of tibia ash

On day 42, two birds from each group weighing close to the average weight of that pen were randomly selected and slaughtered. The left tibia from each slaughtered bird was removed to measure the percentage of ash at 42 days of age. After separating the meat and soft tissues from each tibia, it was weighed. Tibias were defatted by soaking in ethyl alcohol for 48 h and again in ethyl ether for 48 h and then dried to a constant weight using a drying oven at 105°C for 24 h. Then they were ashed in a muffle furnace at 550°C for 12 h (Hamdi et al., 2015).

### 2.6 Economic evaluation

For economic analysis, the ratio of selling price to diet price was obtained and compared for all 12 treatments using the following formula:

$$\mathrm{ratio}\;\mathrm{of}\;\mathrm{selling}\;\mathrm{price}\;\mathrm{to}\;\mathrm{diet}\;\mathrm{price}\;=\;\frac{\mathrm{the}\;\mathrm{price}\;\mathrm{of}\;\mathrm{one}\;\mathrm{kilogram}\;\mathrm{of}\;\mathrm{live}\;\mathrm{weight}\;\mathrm{of}\;\mathrm{chickens}\;\mathrm{at}\;\mathrm{the}\;\mathrm{time}\;\mathrm{of}\;\mathrm{sale}\times \mathrm{final}\;\mathrm{weight}\;\mathrm{of}\;\mathrm{chickens}\;\mathrm{of}\;\mathrm{each}\;\mathrm{treatment}}{\mathrm{total}\;\mathrm{feed}\;\mathrm{intake}\;\mathrm{of}\;\mathrm{each}\;\mathrm{treatment}\times \mathrm{the}\;\mathrm{price}\;\mathrm{of}\;\mathrm{one}\;\mathrm{kilogram}\;\mathrm{of}\;\mathrm{feed}\;\mathrm{in}\;\mathrm{all}\;\mathrm{treatments}}$$

### 2.7 Statistical analysis

The data were analysed based on a completely randomized design in a factorial arrangement of 3 × 2 × 2 (three levels of SM, two levels of Ca and two levels of Phy) by the general linear model procedure of SAS 9.4 (2012) software. Results are shown as means ± standard error of the mean. The means were compared by Tukey's test (*p* < 0.05).

## 3 RESULTS

### 3.1 Growth performance

The main and interaction effects of different levels of SM, Ca and Phy on the DAWG, DFI and FCR are shown in Tables 4 and 5, respectively. The results reveal that the DAWG of broilers fed with diets containing 10% SM was significantly higher than broilers fed with diets containing 20% SM during grower, finisher and whole phases (*p* < 0.05). During finisher period, broilers fed with 0.2% higher than standard Ca levels showed higher DAWG and DFI than those fed with diets containing standard level of Ca (*p* < 0.05). The use of Phy (FTU/kg diet) in the finisher diets significantly increased the DAWG of broilers (*p* < 0.05). Regarding DFI, in the finisher and whole periods, the lowest DFI was observed in the groups consuming 20% SM, which was significantly lower than the groups consuming 10% SM (*p* < 0.05), but it was not significantly different from the treatment without SM (*p* > 0.05). Like DAWG, the highest DFI was observed in chickens receiving more Ca (0.2% higher than standard) and Phy. During the finisher phase, the lowest FCR was obtained in the treatments without SM, which was significantly different from the treatment containing 20% SM (*p* < 0.05). This was despite the fact that during the whole phase, the FCR of broilers in the treatment without SM was significantly lower than other groups (*p* < 0.05). With respect to the two‐way interaction effects, the highest DAWG and DFI during the finisher and whole periods were observed in the treatment of 10% SM × 0.2% Ca higher than standard (*p* < 0.05), which was similar to the treatments without SM × standard Ca and without SM × 0.2% Ca higher than standard (*p* > 0.05). In addition, the highest DAWG was obtained in treatment Phy × 0.2% Ca higher than standard (*p* < 0.05), which was similar to treatments Phy × standard level of Ca and without Phy × 0.2% Ca higher than standard (*p* > 0.05). Three‐way interaction effects were not significant for all parameters (*p* > 0.05).

**TABLE 4:** Main effects of various proportions of sesame meal (SM) replacing soybean meal (SBM), calcium (Ca) and phytase (Phy) on the performance of broilers during the grower, finisher and whole phases.

Treatments	Grower (11–24 days of age)			Finisher (25–42 days of age)			Whole phase (11–42 days of age)		
Main effects	DAWG (g/bird/d)	DFI (g/bird/d)	FCR (g:g)	DAWG (g/bird/d)	DFI (g/bird/d)	FCR (g:g)	DAWG (g/bird/d)	DFI (g/bird/d)	FCR (g:g)
SM (%)									
0	50.77^ab^	75.55	1.50	82.58^a^	137.34^ab^	1.66^b^	68.80^a^	110.09^ab^	1.60^c^
10	51.57^a^	78.32	1.52	79.85^ab^	139.53^a^	1.75^ab^	67.51^ab^	112.71^a^	1.67^ab^
20	45.83^b^	72.45	1.59	73.46^c^	130.01^b^	1.77^a^	60.86^c^	104.78^b^	1.72^a^
*p* Value	0.044	0.088	0.078	<0.0001	0.021	0.014	<0.0001	0.020	<0.0001
SEM	1.72	1.83	0.026	1.14	2.44	0.02	1.20	1.96	0.01
Ca (%)									
Standard	49.89	76.07	1.53	76.31^b^	132.34^b^	1.74	64.74	107.70	1.66
0.2 higher than the standard	48.96	74.80	1.53	80.95^a^	138.92a	1.71	66.71	110.69	1.66
*p* Value	0.644	0.551	0.688	0.009	0.023	0.437	0.164	0.194	0.795
SEM	1.40	1.50	0.026	0.93	1.99	0.02	0.98	1.60	0.01
Phy (FTU/kg diet)									
0	48.57	74.53	1.55	76.88^b^	132.78^b^	1.73	64.49	107.17	1.66
700	50.28	76.35	1.53	80.38^a^	138.47^a^	1.72	66.96	111.21	1.66
*p* Value	0.394	0.395	0.612	0.010	0.049	0.809	0.084	0.081	1.00
SEM	1.40	1.50	0.220	0.93	1.99	0.02	0.98	1.60	0.01

*Note*: Means within a column without a common superscript (^a–c^) significantly differ (*p* < 0.05).

Abbreviations: DAWG, daily average body weight gain; DFI, daily feed intake; FCR, Feed conversion ratio; SEM, Standard error of mean.

**TABLE 5:** Interaction effects of various proportions of sesame meal (SM) replacing soybean meal (SBM), calcium (Ca) and phytase (Phy) on the performance of broilers during the grower, finisher and whole phases.

Treatments		Grower (11–24 days of age)			Finisher (25–42 days of age)			Whole phase (11–42 days of age)		
Interaction effects		DAWG (g/bird/d)	DFI (g/bird/d)	FCR (g:g)	DAWG (g/bird/d)	DFI (g/bird/d)	FCR (g:g)	DAWG (g/bird/d)	DFI (g/bird/d)	FCR (g:g)
SM (%) × Ca (%)	
0	Standard	52.84	77.01	1.46	83.87^ab^	139.87^abc^	1.66	70.30^ab^	112.37^abc^	1.59
10	Standard	49.48	75.51	1.53	74.94^cd^	131.63^bc^	1.76	63.77^bc^	107.01^abc^	1.68
20	Standard	47.34	75.71	1.60	70.11^d^	125.51^c^	1.79	60.15^d^	103.72^c^	1.72
0	0.20 higher than the standard	48.70	74.09	1.54	81.29^abc^	134.82^abc^	1.66	67.31^abcd^	107.81^abc^	1.60
10	0.20 higher than the standard	53.87	81.13	1.51	84.77^a^	147.43^a^	1.73	71.25^a^	118.42^a^	1.66
20	0.20 higher than the standard	44.32	69.19	1.57	76.81^c^	134.50^ab^	1.75	61.58^cd^	105.84^bc^	1.73
*p* Value		0.175	0.066	0.335	0.001	0.013	0.927	0.013	0.021	0.850
SEM		2.43	2.59	0.03	1.61	3.45	0.38	1.71	2.78	0.02
SM (%) × Phy (FTU/kg diet)										
0	0.00	49.47	75.15	1.54	81.28	134.45	1.65	67.36	108.14	1.60
10	0.00	51.93	78.64	1.52	77.65	137.73	1.78	66.39	111.88	1.68
20	0.00	44.32	69.80	1.57	71.72	126.16	1.76	59.73	101.51	1.70
0	700	52.08	75.95	1.46	83.88	140.24	1.67	70.25	112.04	1.59
10	700	51.42	78.00	1.53	82.06	141.32	1.72	68.63	113.56	1.65
20	700	47.34	75.09	1.60	75.20	133.85	1.78	62.00	108.05	1.74
*p* Value		0.732	0.499	0.486	0.852	0.838	0.486	0.977	0.683	0.330
SEM		2.43	2.59	0.03	1.61	3.45	0.03	1.71	2.78	0.02
Ca (%) × Phy (FTU/kg diet)										
Standard	0.00	47.98	74.47	1.55	73.18^d^	127.34	1.75	62.15	104.21	1.68
Standard	700	51.80	74.59	1.51	79.44^abc^	137.33	1.73	67.32	111.19	1.65
0.2 higher than the standard	0.00	49.16	77.68	1.54	80.59^ab^	138.23	1.71	66.84	110.14	1.65
0.2 higher than the standard	700	48.76	75.02	1.55	81.33^a^	139.61	1.72	66.59	111.24	1.67
*p* Value		0.294	0.515	0.313	0.041	0.132	0.684	0.058	0.201	0.198
SEM		1.99	2.12	0.03	1.31	2.81	0.03	1.39	2.27	0.02
				*p* Value						
SM (%) × Ca (%) × Phy (FTU/kg diet)		0.716	0.676	0.843	0.631	0.713	0.689	0.986	0.825	0.384

*Note*: Means within a column without a common superscript (^a–d^) significantly differ (*p* < 0.05).

Abbreviations: DAWG, daily average body weight gain; DFI, daily feed intake; FCR, feed conversion ratio; SEM, standard error of mean.

### 3.2 Carcass characteristics

The results of carcass characteristics percentage at 42 days of age are presented in Table 6. Investigating the main effects of SM showed that chickens receiving diets containing 20% SM had a lower percentage of breast weight compared to other treatments (*p* < 0.05). In addition, the increase of SM in the diet caused a significant increase in the heart weight of chickens (*p* < 0.05). This was despite the fact that the birds consuming diets containing 20% SM compared to the 10% SM had a higher percentage of liver weight (*p* < 0.05). In relation to the main effect of Ca, increased Ca levels reduced heart weight (*p* < 0.05). No significant interactions between other factors were observed (*p* > 0.05).

**TABLE 6:** Main and interaction effects of various proportions of sesame meal (SM) replacing soybean meal (SBM), calcium (Ca) and phytase (Phy) on the carcass and organs characteristics (%) of broilers at 42 days of age.

Treatments			Carcass characteristics			
Main effects	Carcass	Breast	Thigh	Wings	Liver	Heart
SM (%)
0	71.44	27.64^a^	18.97	8.43	2.15^ab^	0.50^b^
10	70.50	27.08^ab^	18.69	8.26	2.03^b^	0.55^ab^
20	68.40	24.87^c^	18.96	8.78	2.24^a^	0.57^a^
*p* Value	0.115	0.007	0.935	0.271	0.020	0.029
SEM	1.03	0.50	0.67	0.23	0.05	0.01
Ca (%)						
Standard	69.78	26.45	18.53	8.48	2.18	0.56^a^
0.2 higher than the standard	70.44	26.61	19.22	8.50	2.10	0.51^b^
*p* Value	0.558	0.778	0.338	0.948	0.229	0.021
SEM	0.84	0.40	0.50	0.18	0.04	0.01
Phy (FTU/kg diet)						
0	70.71	26.54	19.32	8.40	2.10	0.52
700	69.51	26.53	18.43	8.58	2.18	0.56
*p* Value	0.325	0.986	0.210	0.515	0.212	0.072
SEM	0.84	0.40	0.50	0.18	0.04	0.01
			*p* Value			
Interaction effects						
SM (%) × Ca (%)	0.161	0.697	0.129	0.217	0.243	0.585
SM (%) × Phy (FTU/kg diet)	0.727	0.437	0.700	0.338	0.273	0.686
Ca (%) × Phy (FTU/kg diet)	0.760	0.118	0.683	0.938	0.505	0.815
SM (%) × Ca (%) × Phy (FTU/kg diet)	0.195	0.084	0.346	0.774	0.622	0.917

*Note*: Means within a column without a common superscript (^a–c^) significantly differ (*p* < 0.05).

Abbreviations: SEM, standard error of mean.

### 3.3 Serum parameters and tibia ash

The data related to the main and interaction effects of SM, Ca and Phy on the serum biochemical profile and bone ash percentage are shown in Table 7. The results showed that by increasing the level of SM in the diets, the amount of serum Ca decreased. So that the highest amount of serum Ca was observed in the treatment without SM, which was similar to the treatment containing 10% SM (*p* > 0.05), but it was significantly different from the treatment containing 20% SM (*p* < 0.05). No significant interactions were observed (*p* > 0.05). The amount of tibia ash was not affected by the treatments (*p* > 0.05).

**TABLE 7:** Main and interaction effects of various proportions of sesame meal (SM) replacing soybean meal (SBM), calcium (Ca) and phytase (Phy) on the serum parameters and tibia ash of broilers at 42 days of age.

Treatments							
Main effects	Triglyceride (mg/L)	Total cholesterol (mg/L)	HDL (mg/L)	LDL (mg/L)	Ca (mg/L)	P (mg/L)	Tibia ash (%)
SM (%)							
0	110.90	116.15	46.65	46.75	10.69^a^	6.53	40.50
10	111.25	122.45	51.65	47.95	10.32^ab^	6.35	33.33
20	116.30	128.15	52.35	51.50	10.17^b^	6.41	36.39
*p* Value	0.712	0.086	0.152	0.158	0.043	0.718	0.435
SEM	5.17	3.74	2.22	1.78	0.14	0.15	3.97
Ca (%)							
Standard	116.16	124.33	49.46	50.20	10.34	6.32	35.43
0.2 higher than the standard	109.46	120.16	50.96	47.26	10.44	6.55	38.55
*p* Value	0.267	0.339	0.561	0.160	0.530	0.215	0.480
SEM	4.22	3.05	1.81	1.45	0.12	0.13	3.23
Phy (FTU/kg diet)							
0	113.73	125.66	50.90	49.60	10.43	6.46	38.18
700	111.90	118.83	49.53	47.86	10.35	6.41	35.78
*p* Value	0.760	0.120	0.597	0.404	0.610	0.786	0.574
SEM	4.22	3.05	1.81	1.45	0.12	0.13	3.23
			*p* Value				
Interaction effects							
SM (%) × Ca (%)	0.056	0.670	0.924	0.458	0.755	0.094	0.330
SM (%) × Phy (FTU/kg diet)	0.208	0.951	0.766	0.545	0.339	0.425	0.560
Ca (%) × Phy (FTU/kg diet)	0.300	0.544	0.887	0.699	0.906	0.202	0.960
SM (%) × Ca (%) × Phy (FTU/kg diet)	0.727	0.897	0.810	0.592	0.370	0.529	0.960

*Note*: Means within a column without a common superscript (^a,b^) significantly differ (*p* < 0.05).

Abbreviations: HDL, high‐density lipoprotein cholesterol; LDL, low‐density lipoprotein cholesterol; P, phosphorus: SEM, standard error of mean.

### 3.4 Economic evaluation

The data related to the economic investigation of replacing SBM with SM is shown in Table 8. Considering the price of diet and the selling price of live chickens, it will be more economical to use treatments containing 20% SM.

**TABLE 8:** The ratio of the chickens selling price (each treatment) at the age of 42 days to the price obtained for each diet in the whole period of study.

Treatments			
SM (%)	Ca (%)	Phy (FTU/kg diet)	Selling price: diet price
0	Standard	0	1.44
0	0.2 higher than the standard	0	1.52
0	Standard	700	1.41
0	0.2 higher than the standard	700	1.45
10	Standard	0	1.77
10	0.2 higher than the standard	0	1.67
10	Standard	700	1.74
10	0.2 higher than the standard	700	1.65
20	Standard	0	2.08
20	0.2 higher than the standard	0	2.02
20	Standard	700	2.03
20	0.2 higher than the standard	700	1.84

Abbreviations: Ca, calcium; Phy, phytase; SM, sesame meal.

## 4 DISCUSSION

In accordance with the results of the present experiment, Rahimian et al. (2013) also reported that the use of more than 10% SM in the broilers’ diet caused a significant decrease in the chickens’ body weight. Kaneko et al. (2002) reported that the body weight of chickens fed with a diet containing 14% SM decreased considerably. It has been reported that the use of 12% SM reduced the growth of broilers (Farran, 2000). In the current study, the results in the grower period showed that the DAWG of broilers fed with a diet including 20% SM was notably lower than that of broilers fed with a diet comprising 10% SM. On the other hand, Rao et al. (2008) stated that the use of SM by 54% and 31% did not cause a difference in the weight of chickens at the age of 21 and 42 days, respectively. Yamauchi et al. (2006) also observed that the final weight and DWG of chickens fed with a diet containing 20% SM were not significantly different from the groups receiving 10% SM and control. In the present experiment, the highest DWG was observed in treatments containing 10% SM × 0.2% higher than the standard Ca. Kim et al. (2017) attributed the beneficial effects of Phy to more free available P than phytate‐phosphorus, which could reduce its anti‐nutritional impact. This improved utilization of phytate‐phosphorus ameliorates the utilization of energy and other nutrients such as amino acids and minerals in the diet (Attia et al., 2016). In a study conducted by Cheva‐Isarakul and Tangtaweewipat (1993), 8% SM in the diet of chickens did not have a significant effect on feed consumption. When using different levels of SM, lysine and Phy in the diet of laying hens, Baghel et al. (2020) reported that birds consuming diets with 10% SM had higher feed intake than the treatments fed with 0% and 20% SM. This was despite the fact that the FCR was not affected by different levels of SM. In the present study, the use of SM worsened the FCR. Sina et al. (2014) and Rezaeipour et al. (2016) reported that Japanese quails reduce feed intake when fed with dietary SM. The reduction in feed intake with greater dietary amounts of SM has been attributed to the presence of bitter substances in it (Baghel et al., 2020). The reason for the different results can be the level of SM used in the diet, its chemical composition, meal production method (mechanical or solvent) the use or non‐use of Phy and its level, bird strain and other nutritional factors.

Al Harthi and El‐Deek (2009) reported that carcass characteristics were not affected by different levels of SM in the diets. However, increasing the level of SM by 15% led to a decrease in the relative weight of the breast. Agbulu et al. (2010) showed that carcass weight, liver and heart of birds fed with different levels of SM (0%, 3%, 6%, 9% and 12%) were not affected by the treatments. Another study showed that when different levels of SM (0%, 4.7%, 9.4% and 14.1%) were used in the broiler chickens’ diets, birds consuming 9.40% and 14.10% SM showed lower breast and thigh weights (Kaneko et al., 2002). Ahmed et al. (2004) also reported that the use of different levels of Phy had no significant effect on the weight percentage of carcass components. In agreement with our experiment, Rahimian et al. (2013) reported that the relative weight of liver increased significantly with increasing levels of SM. They attributed this increment to an increase in anti‐nutritional substances in the diet and an increase in its activity, because the liver is mainly responsible for neutralizing toxic substances in the body.

Rao et al. (2008) reported that the inclusion of SM levels in the diet had no significant effect on the serum Ca and P concentrations. In the current study, blood serum Ca concentration of chickens consuming diets containing 20% SM was significantly lower than the groups consuming diets containing 0% SM. At the same time, the use of 10% SM in the diet had no effect on serum Ca concentration, which is probably due to insufficient phytate to affect blood Ca levels. Rahimian et al. (2013) reported that birds fed with a diet without SM had the lowest levels of Ca and P in their blood. They stated that Phy supplementation had a notable effect on the amount of Ca and P in tibia, whereas the level of Ca in the broilers’ blood was not affected. Ghazvinian et al. (2016) did not observe any significant effect on the triglycerides, cholesterol, HDL and LDL when using different levels of SM. In a study conducted on laying hens, the inclusion of SM at a level of 20% in the diet decreased the concentration of LDL but increased the concentration of HDL in the serum (Baghel et al., 2020). In agreement with our study, Rao et al. (2008) reported that the inclusion of 15.5 of SM in the diet had no effect on the weight of tibia bone ash compared to the diet containing 0% SM, but increasing the concentration of SM to 31% resulted in the reduction of tibia bone ash, and with increasing the concentration of SM in the diet decreased the relative weight, breaking strength and ash content of tibia. This reduction in bone mineralization and tibia weight after increasing the amount of SM is due to the phytic acid present in it, which reduces the availability of Ca and P. In agreement with the results of the present experiment, Ghavidel‐Heydari et al. (2021) stated that due to the high cost and low availability of SBM in the country, it is recommended to use SM in the diet of Japanese quails to overcome the lack of SBM and reduce feed costs. In our study, diets containing 20% SM were more economical.

## 5 CONCLUSIONS

In conclusion, the use of 10% SM instead of SBM and 0.2% higher than the standard amount of Ca did not show any negative effect on the performance of birds. The use of Phy in diets containing SM improved the performance of broilers. On the other hand, the use of 20% SM reduced the cost of diets.

## AUTHOR CONTRIBUTIONS

All authors are either employed by, or associated with, a government agency or university, whose primary function is research and education.

## CONFLICT OF INTEREST STATEMENT

The authors declare that there are no known conflicts of interest.

## FUNDING INFORMATION

Ferdowsi University of Mashhad, Project Number 49955

## ETHICS STATEMENT

All procedures were approved by the Animal Care and Use Committee of the Ferdowsi University of Mashhad, Mashhad, Iran.

### PEER REVIEW

The peer review history for this article is available at https://publons.com/publon/10.1002/vms3.1379.

## Data Availability

The data that support the findings of this study are available from the corresponding author upon reasonable request.
